# Acute Gastric Dilatation Resulting in Gastric Emphysema Following Postpartum Hemorrhage

**DOI:** 10.1155/2012/230629

**Published:** 2012-06-24

**Authors:** Suhail Aslam Khan, Edmond Boko, Haseeb Anwar Khookhar, Sheila Woods, A. H. Nasr

**Affiliations:** ^1^Department of Surgery, Our Lady of Lourdes Hospital, Drogheda, County Louth, Ireland; ^2^Department of Radiology, Our Lady of Lourdes Hospital, Drogheda, County Louth, Ireland

## Abstract

Acute gastric dilatation is a rare entity, with varying aetiologies the majority of which are benign. Delay in diagnosis and treatment could result in sequelae such as gastric emphysema (pneumatosis), emphysematous gastritis, gangrene, and perforation. Gastric emphysema as a result of a benign nongangrenous condition such as gastroparesis, adynamic ileus can be successfully managed conservatively. Here, we present an interesting case of acute gastric dilatation resulting in gastric emphysema following massive postpartum hemorrhage.

## 1. Introduction

Acute gastric dilatation is a rare entity, with varying aetiologies, the majority of which are benign. Prompt recognition and appropriate management are essential to prevent sequelae such as gastric emphysema (pneumatosis), emphysematous gastritis, gangrene, and perforation [1–4]. Like other sequelae of acute gastric dilatation, the development of gastric emphysema may also reflect other intra-abdominal pathology, and its presence can suggest gangrenous changes of the stomach or colon and, therefore, represents a surgical emergency [1–3]. However, gastric emphysema can also occur as a result of a benign, nongangrenous condition, such as gastroparesis and adynamic illeus and can be successfully managed conservatively [5–7]. Here we are presenting an interesting case of acute gastric dilatation resulting in gastric emphysema following acute management of postpartum hemorrhage.

## 2. Case Report

A 40-year-old African gravid 3/para 3 woman was admitted to the intensive care unit (ICU) following a caesarian section for placental abruption and fetal distress. She was booked for antenatal care at approximately 34 weeks of pregnancy with antenatal care prior to that done in Africa for this pregnancy. Her initial assessment was satisfactory (Tables 1 and 2), but in the background history, it was noted that she was suffering from constipation and gestational diabetes mellitus in her last pregnancy (Table 2) and was poorly compliant with insulin therapy.

She underwent emergency C-section after she presented with antepartum haemorrhage, and intraoperative findings were a low-lying placenta with large retroplacental clot with abruption. Postoperatively, she continued to bleed per vagina in ICU and received 7 units of red cell concentrate (RCC), 4 units of plasma, 4 pools of platelets, and an oxytocin infusion. Despite treatment, she continued to bleed and was transferred back to theatre, where a uterine tamponade balloon was successfully inserted. Her total blood loss was estimated in excess of 3 litres.

**Figure 1:**
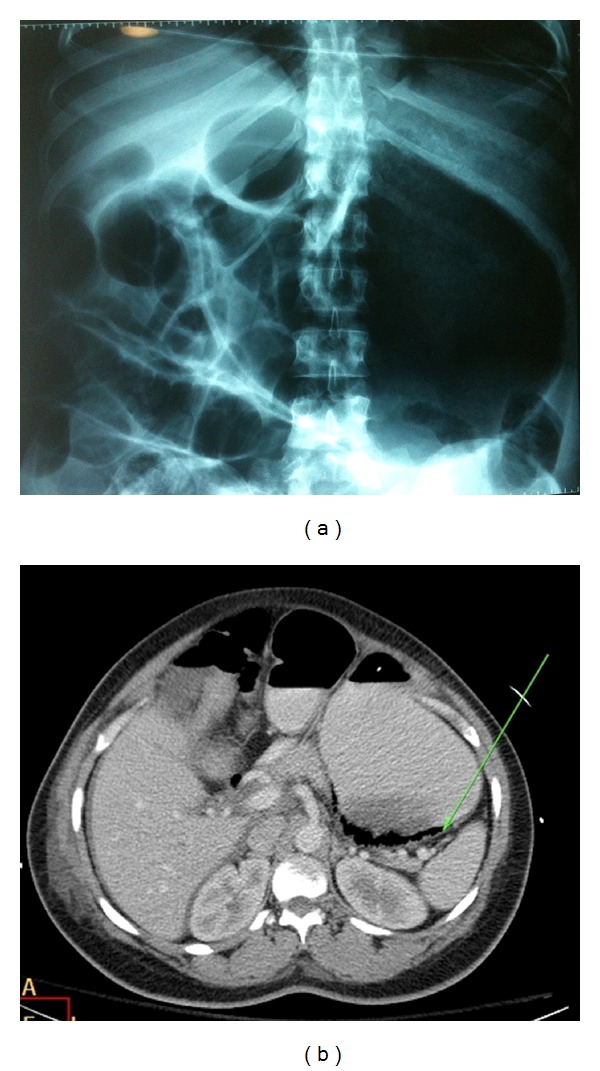
Plain film of abdomen and CT abdomen showing gastric dilatation of stomach and emphysema (arrow).

**Figure 2:**
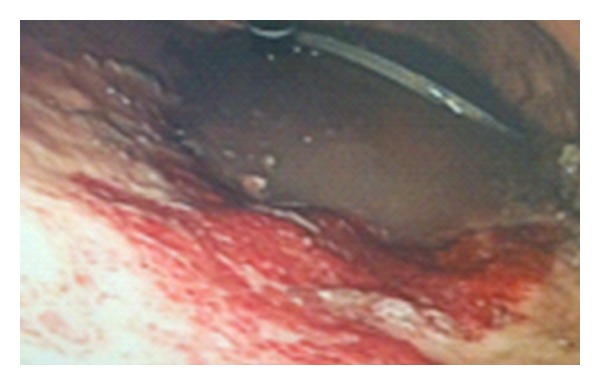
Upper GI endoscopy: showing oedamatous beefy red mucosa along the greater curvature of stomach.

On the same evening, she began to complain of abdominal pain. On examination, her vital signs were within the normal range. Abdominal examination revealed a distended abdomen with tenderness in the epigastrium and left upper quadrant and was tympanic to percussion over the epigastric region. There was no guarding or rigidity noted. An abdominal X-ray showed significant gastric distension with diffusely noted gas pattern consistent with gastric emphysema (Figure 1). CT confirmed air in the stomach wall, with moderate abdominal and pelvic fluid, which was attributed to her earlier C-section (Figure 1). An urgent upper GI endoscopy was arranged which revealed a normal oesophagus, food in the stomach, and, on washing, revealed an oedematous beefy red, friable mucous-coated stomach mucosa extending over greater curvature from midstomach towards the fundus (Figure 2). Following consultation with the upper GI unit in a tertiary referral centre, this patient was managed conservatively with gastric drainage via nasogastric tube, broad-spectrum antibiotic cover, oral sucralfate, and total parenteral nutrition with close monitoring in ICU.

**Figure 3:**
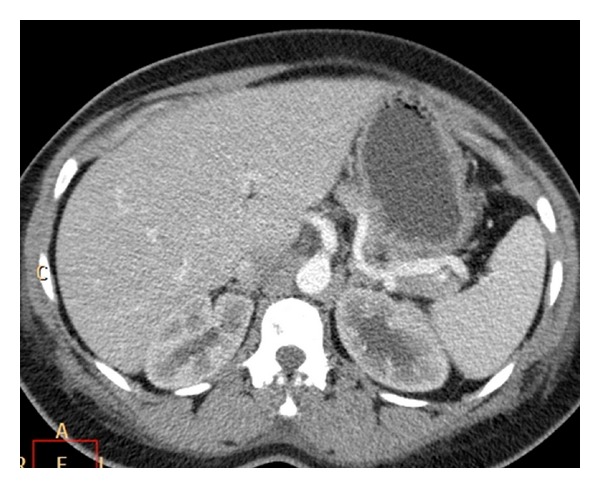
Follow-up CT abdomen after a week shows resolution of gastric emphysema.

Her further clinical course was uneventful, and she responded very well to conservative management. A repeat CT, one week later, showed absence of gastric wall gas or free air (Figure 3). The large and small bowel loops appeared normal, and she was discharged home.

## 3. Discussion

Acute gastric dilation was first described by Powell et al. in 2003 [1], and it can be as a result of eating disorders, hemorrhage/trauma resuscitation, volvulus, medications, electrolyte abnormalities, infections, superior mesenteric artery syndrome, diabetes mellitus and slow gut transits causing chronic constipation [1–11]. It can have devastating consequences and has a reported mortality rate of 80% to 100% as a consequence of gastric necrosis and perforation [1–4].

Acute gastric dilatation can result in gastric emphysema, emphysematous gastritis, and ischemic necrosis. Ischemic necrosis, in the case of gastric dilatation, is postulated to be due to venous insufficiency [1, 5, 6]. Pressure in the stomach lumen must be >14 mm Hg to exceed gastric venous pressure and lead to ischemia [1, 3, 4]. Gastric emphysema is also a rare finding with only a few cases reported in the literature. Gastric emphysema resulting from a violation of mucosal integrity followed by forceful entry of air between the gastric layers is called noninfectious gastric emphysema [5, 6]. The other extremely rare condition that was first described by Fraenkel in 1889 as emphysematous gastritis is due to infection [9–11]. The most commonly involved microorganisms are *streptococci*, *Escherichia coli*, *Pseudomonas aeruginosa*, *Clostridium perfringens*, and *Staphylococcus aureus* [10].

Gastric emphysema secondary to mechanical aetiologies is far more common than primary gastric emphysema. Causes include obstruction, trauma, and rupture of pulmonary bullae, enteric tube placement, and upper endoscopic procedures [1–8]. In these cases, the intraluminal gas dissects into the gastric wall through a mucosal tear or defect. The tear usually results from increased intraluminal pressure secondary to an obstruction or as a direct result of trauma. The reported mortality in this group, although lower than the mortality associated with gastric emphysema, is still high at 6% to 41% [3–5]. While other authors describe gastric emphysema secondary to bowel or gastric outlet obstruction, Cho et al. highlighted the association of gastric emphysema causing adynamic ileus with postpartum hemorrhage, but gastric emphysema specifically associated with caesarian section and intrapartum hemorrhage has not been reported [7].

Clinically, patients with infectious gastric emphysema appear septic and complain of fever, chills, and abdominal pain [2, 9–11]. These signs and symptoms could be the main differentiating features separating infectious gastritis from symptoms of acute gastric dilation resulting in gastric emphysema, which can be initially vague with progressive abdominal distention and accompanying pain [9–11]. Plain abdominal radiographs or abdominal computed tomography are diagnostic. In the majority of cases, emphysema occurs along the greater curvature of the stomach. The lesser curvature and pyloric regions of the stomach tend to be spared [2, 5, 12, 13]. In a pictorial essay, Johnson et al. presented a series of cases of gastric emphysema. They advocated the use of CT to guide management and to assist in the identification of associated extragastric pathologies [13].

Early endoscopy in the stable patient may be of use, particularly if the CT scan suggests gastric or esophageal involvement. Where diagnosis is emphysematous gastritis, gastroscopy and biopsy are advocated to facilitate a diagnosis, which can show evidence of submucosal abscesses or exudative gastritis [14, 15]. In the above case, where the aetiology is obscure, the endoscopic findings of diffuse inflammation with diffusely friable, oedematous mucosa with mucopurulent exudates and rapid resolution of signs and symptoms may suggest a transient ischemia [5]. Because of transient ischemia, the mucosa may be injured or compromised thus allowing air to penetrate and dissect into the gastric wall. In these patients, nonoperative management and nasogastric decompression resulted in resolution of emphysema in 72 hours [5, 6].

Treatment focuses on early diagnosis, broad-spectrum antibiotic cover, nasogastric decompression of the stomach, and total parenteral nutrition, thus halting the vascular congestion and ensuing ischemia [3–5]. When considering gastric emphysema due to mechanical causes, clinical judgment should be carefully applied when determining which patients require surgical exploration and which can be observed safely. Selective non-operative management has been used successfully in the setting of secondary gastric emphysema, with nongangrenous gastric and esophageal emphysema [5]. Delayed perforation or bleeding is still possible, even following decompression. Surgical exploration is mandated in the presence of instability, perforation or for other indications, such as associated small bowel obstruction or ischemia of the small bowel [1, 4, 7].

In our case, it is reasonable to presume that multiple factors have played a role in the development of acute gastric dilatation and gastric emphysema. This patient's co-morbidities, gestational diabetes with poor diabetic control leading to gastroparesis, and chronic constipation are likely to have been a factor in the development of acute gastric dilatation and subsequently gastric emphysema. It is also likely that other factors can potentially aggravate the condition in this patient such as increase in intra-abdominal pressure because of gastric dilatation and acute massive haemorrhage, leading to transient poor perfusion of the gastrointestinal tract. Also the use of opiates for pain relief can further potentiate depression of gut motility. From the above case report and discussion, it is clear that the diagnosis and treatment of underlying cause of gastric emphysema are most important for a favourable outcome then simple gastric decompression.

## Figures and Tables

**Table 1:** Antenatal history.

Date of booking	22/03/2011
LMP, EDD, gestation	28/7/10, 4/5/11, 33 wks, and 6 days
Cycle length	28, regular
Maternal risk category	Low risk
Allergies	Flagyl, Chloroquine
Drugs in pregnancy	Antimalarial tabs and folic acid
Booking BP, pulse	126/76, 74/min
Smoking, alcohol	None
Medical conditions	H/O essential HTN on med for 6 months in 2010, Rec UTIs, gestational diabetes in previous two pregnancies, constipation
Family history	Sickle cell anemia, DM type 1
Surgical history	Appendicectomy

Ultrasound	

EDD by LMP	4/5/11
EDD by USS	4/5/11
BPD	87.8 mm
ABD circumf.	331.3
Placental site	Upper ant
Wt differential	454 gms
Growth centile	>90
Presentation	Cephalic
Fetal cardiac activity	Present
Head circumf.	306.7
Femur length	77 mm
Fetal wt	3113 gms
CTG	Satisfactory

**Table 2:** Record of previous pregnancies.

Year	2001, Nigeria
Gestation	39 wks
Antenatal problems	No record
Mode of delivery	Spontaneous, vertex, hospital
Perineal problems	Infected episiotomy
Outcome	Live birth, 3690 gms male

Year	2003, Ireland

Gestation	40 wks
Antenatal problems	Gestational diabetes
Onset	Spontaneous
Mode of delivery	Spontaneous, vertex, hospital
Outcome	Live birth, 3890 gms female

Year	2006, Ireland

Gestation	40 wks
Antenatal problems	Gestational diabetes, insulin given
Onset	Induced
Mode of delivery	Spontaneous, vertex, hospital
Outcome	Live birth, 3790 gms male
Neonatal problems	Yes; SCBU for BSL monitoring for 3 days
